# Clinical Features of Pulmonary Nocardiosis and Diagnostic Value of Metagenomic Next-Generation Sequencing: A Retrospective Study

**DOI:** 10.3390/pathogens14070656

**Published:** 2025-07-02

**Authors:** Yanbin Chen, Hailong Fu, Qiongfang Zhu, Yalu Ren, Jia Liu, Yining Wu, Jie Xu

**Affiliations:** 1 Department of Pulmonary and Critical Care Medicine, The First Affiliated Hospital of Soochow University, 899#, Pinghai Road, Suzhou 215031, China; chenyanbin@suda.edu.cn; 2 Center for Clinical Laboratory, The First Affiliated Hospital of Soochow University, 899#, Pinghai Road, Suzhou 215031, China; fuhailong@suda.edu.cn (H.F.); zhuqiongfang1987@suda.edu.cn (Q.Z.); renyalu1989@suda.edu.cn (Y.R.); wuyining@suda.edu.cn (Y.W.); 3 Department of Medical, Nanjing Dinfectome Technology Inc., Nanjing 210032, China; jia.liu81039@dinfectome.com

**Keywords:** *Nocardia* infection, clinical characteristics, detection performance, treatment

## Abstract

Pulmonary nocardiosis (PN) is a rare, opportunistic, and potentially life-threatening infection, especially in disseminated cases. This retrospective study aimed to characterize the clinical features of PN and assess the diagnostic utility of metagenomic next-generation sequencing (mNGS). We reviewed data from 19 patients diagnosed with PN between September 2019 and August 2022, including 3 with disseminated disease. Common symptoms included fever, cough, and sputum production, while chest imaging frequently revealed nodules, consolidations, exudates, cavities, and pleural effusions. The sensitivity of mNGS for detecting *Nocardia* was significantly higher than that of culture (100% vs. 36.84%, *p* < 0.001). mNGS successfully identified *Nocardia* species and co-infected pathogens. The most common species was *Nocardia farcinica*. Four PN cases were co-infected with *Rhizomucor pusillus*, *Cryptococcus neoformans*, *Lichtheimia ramosa*, and *Aspergillus* spp. Eighteen patients (94.7%) received trimethoprim-sulfamethoxazole (TMP-SMZ). Sixteen cases (84.2%) were improved or cured. Misdiagnosis is common due to the nonspecificity of clinical and imaging presentations of pulmonary nocardiosis. The timely combination of mNGS represents a promising approach to enhance the diagnosis of pulmonary nocardiosis and inform targeted antimicrobial therapy. TMP-SMZ is the first line of treatment.

## 1. Introduction

Nocardiosis is a disease caused by *Nocardia*, which was initially described by Edmond Nocard in 1888 [1,2]. *Nocardia* is a genus of weakly acid-fast, Gram-positive filamentous bacilli widely distributed in soil, decomposing vegetation, organic matter, and both freshwater and saltwater ecosystems [3]. As opportunistic pathogens, *Nocardia* species primarily cause infections in immunocompromised individuals, with clinical manifestations ranging from localized lesions to life-threatening disseminated disease. The first human case of nocardiosis was reported by Epinger in 1891 [4]. The most commonly clinical species are *Nocardia nova*, *Nocardia farcinica*, *Nocardia cyriacigeorgica*, *Nocardia brasiliensis*, and *Nocardia abscessus*. Epidemiological data are limited in China, while in the United States, an estimated 500 to 1000 new cases occur annually (https://www.cdc.gov/nocardiosis/hcp/clinical-overview/?CDC_AAref_Val=https://www.cdc.gov/nocardiosis/health-care-workers/index.html, accessed on 9 March 2025). Pulmonary nocardiosis is the most prevalent clinical form and may also involve extrapulmonary sites such as the skin, subcutaneous tissue, and central nervous system [5]. Pulmonary nocardiosis, especially disseminated cases, is potentially fatal and deserves attention.

The incidence of nocardiosis has increased in recent years [6], partly due to the rising use of long-term glucocorticoids and other immunosuppressive therapies, as well as improvements in diagnostic methods, particularly metagenomic next-generation sequencing (mNGS). mNGS offers high diagnostic sensitivity, a shorter turnaround time, and the ability to identify nocardiosis in addition to culture methods [7]. However, most published studies on the use of mNGS in nocardiosis focus on individual cases and lack comprehensive analysis [8,9,10,11]. In this study, we retrospectively analyzed 19 cases of nocardiosis to systematically examine their clinical presentations, diagnostic approaches, treatment strategies, and prognoses. Our aim was to enhance physicians’ awareness of nocardiosis and facilitate its early diagnosis and treatment.

## 2. Materials and Methods

### 2.1. Participants

For this study, 3223 patients with suspected infection admitted to the First Affiliated Hospital of Soochow University between September 2019 to August 2022 were enrolled consecutively. All patients underwent clinical specimen collection for both conventional pathogen testing and mNGS. Nineteen patients were diagnosed with pulmonary nocardiosis and included in this retrospective analysis.

The inclusion criteria were as follows: (1) positive culture for *Nocardia* from sputum specimens; (2) focal tissue of lung, skin, subcutaneous abscess or cerebrospinal fluid, pleural effusion, peripheral blood, bronchoalveolar lavage fluid (BALF) obtained under aseptic operating conditions, confirmed positive for *Nocardia* by common culture, mNGS test, imaging, etc.; and (3) complete clinical information. *Nocardia* species were identified by conventional phenotypic identification methods and mNGS. Criteria for determining disseminated nocardiosis included ≥2 non-adjacent organs clearly infected with *Nocardia* or positive blood culture for *Nocardia*.

Three of the nineteen patients with pulmonary nocardiosis were reported in a retrospective study conducted by our respiratory team from January 2019 to April 2022 [10]. In contrast, the present study focuses on an analysis of pulmonary nocardiosis across the entire hospital from September 2019 to August 2022, aiming to (1) estimate the incidence and characterize clinical features of pulmonary nocardiosis, and (2) compare the positivity rates, diagnostic performance, and clinical impacts of metagenomic next-generation sequencing (mNGS) and culture-based assays for *Nocardia* detection. The difference in study scope is clarified herein for transparency.

The study was conducted in accordance with the Declaration of Helsinki and approved by the Ethics Committee of The First Affiliated Hospital of Soochow University (Approval No. 2018-189). Informed consent was obtained from all participants involved in the study.

### 2.2. Clinical and Sample Collection

Five types of clinical specimens were collected from the 19 patients, including 12 BALF samples, six sputum samples, six blood samples, one pleural effusion sample, one secretion sample, and one skin puncture fluid sample.

Clinical information was retrieved from medical records, including demographics (gender, age), underlying disease, clinical symptoms, imaging results, specimen type, pathogen test results, treatment strategies, and clinical outcomes. Clinical follow-up was performed until hospital discharge or death.

### 2.3. Conventional Microbiological Tests

Qualified sputum or BALF samples were collected from the bedside for a 3-day culture inoculation on Columbia blood agar and chocolate-colored blood agar plates at 35 °C with 5% carbon dioxide. Serological tests, including (1,3)-β-D glucan (BDG) and galactomannan (GM) tests, were used for the diagnosis of fungal infections.

### 2.4. Metagenomic Next-Generation Sequencing and Analysis

Sputum was liquefied by 0.1% dithiothreitol (DTT, Sangon Biotech, Shanghai, China) for 20 min at 56 °C before extraction. Plasma was prepared from blood samples. Cell-free DNA was isolated from plasma with the QIAamp Circulating Nucleic Acid Kit (Qiagen, Hilden, Germany) according to the manufacturer’s protocols. Sputum DNA was extracted using the TIANamp Magnetic DNA Kit (Tiangen, Beijing, China) according to the manufacturer’s protocols. The quantity and quality were assessed using the Qubit (Thermo Fisher Scientific, Austin, TX, USA) and NanoDrop (Thermo Fisher Scientific, Wilmington, DE, USA), respectively.

DNA libraries were prepared using the KAPA Hyper Prep kit (KAPA Biosystems, Wilmington, MA, USA) according to the manufacturer’s protocols. Agilent 2100 Bioanalyzer (Agilent Technologies, Santa Clara, CA, USA) was used for quality control and DNA libraries were 75bp single-end sequenced on Illumina NextSeq 550Dx (Illumina, San Diego, CA, USA).

Raw sequencing data were split by bcl2fastq2 (version 2.20, Illumina, San Diego, CA, USA), and high-quality sequencing data were generated using Trimmomatic (version 0.36) by removing low-quality reads, adapter contamination, duplicated, and shot (length < 36 bp) reads. The human host sequence was subtracted by mapping to the human reference genome (hs37d5) using bowtie2 (version 2.2.6). Reads that could not be mapped to the human genome were retained and aligned with the microorganism genome database for microbial identification by Kraken (version 2.0.7), and for species abundance estimating by Bracken (version 2.5.0). The microorganism genome database contained genomes or scaffolds of bacteria, fungi, viruses, and parasites (download from GenBank release 238, https://ftp.ncbi.nlm.nih.gov/genbank/, accessed on 24 July 2020). Criteria for detection positivity were as follows: (1) at least one species-specific read for the detection of *Mycobacterium*, *Nocardia*, and *Legionella pneumophila*; (2) at least three unique reads were required for other bacteria, fungi, viruses, and parasites; and (3) pathogens were excluded if the ratio of microorganism reads per million of a given sample to NTC was < 10. The background of microbial communities occurring in the normal population was deducted to identify the potential pathogenic microorganisms. 

### 2.5. Statistical Analysis

Statistical analyses were performed on SPSS 22.0 software (IBM, Armonk, NY, USA). Continuous variables data of normal distribution were expressed as the mean ± standard deviation (SD). Categorical variables were expressed as number and percentage (%). A 2 × 2 contingency table was constructed to calculate the sensitivity, specificity, positive predictive value (PPV), negative predictive value (NPV), and diagnostic accuracy for both mNGS and culture methods, using the clinical diagnosis of pulmonary nocardiosis as the reference standard. Group comparisons were made using *Chi*-squared or *Fisher*’s exact test. A *p*-value < 0.05 was considered statistically significant.

## 3. Results

### 3.1. Clinical Characteristics of Patients

Nineteen patients were diagnosed with pulmonary nocardiosis, representing 0.6% (19/3223) of the suspected infection cohort during the study period. The clinical characteristics of these patients are summarized in Table 1. Eleven of the nineteen patients with pulmonary nocardiosis were male. Patients’ age ranged from 29 to 83 with a mean age of 58. Seventeen patients (89.5%) had multiple underlying diseases, including autoimmune disease, pulmonary disease, and hematologic malignancies. The remaining two patients had no underlying disease. Fourteen patients (73.7%) had a history of long-term use of oral glucocorticoids and/or other immunosuppressive therapies.

Among the 19 patients, one case was unintentionally found due to the lung nodular shadow on physical examination without symptoms. The remaining 18 cases had cough and sputum and other discomfort. Thirteen cases had fever (>37.2 °C), including six cases having a temperature above 39 °C. Four cases showed a drop in white blood cell (WBC) levels, while eight cases showed an increase and seven cases were within the normal range. Fourteen cases showed a decrease in lymphocyte count (LY), while two cases showed an increase, and three cases fell within the normal range. Serum albumin (ALB) levels were decreased in 15 patients and normal in the remaining 4 patients (Supplementary Table S1).

Among the 19 patients with pulmonary nocardiosis, there were three cases of the disseminated type. One patient had concurrent pulmonary and bloodstream infection; another had involvement of the lungs, pleura, and bloodstream; and the third had multisite infection affecting the lungs, skin, brain, and eyes. The remaining 16 patients had localized pulmonary nocardiosis without extrapulmonary involvement.

**Figure 1:**
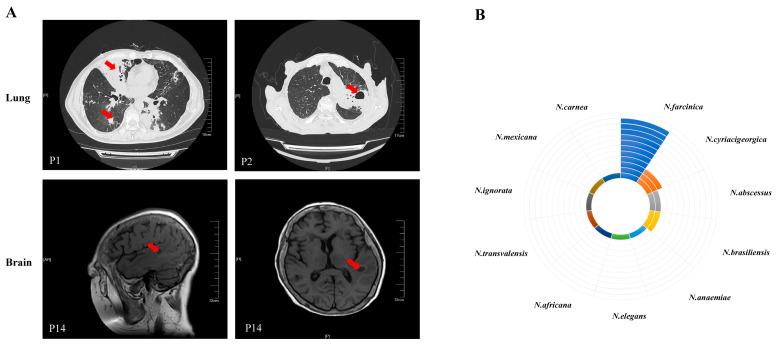
Diagnostic findings in nocardiosis. (**A**) Representative imaging of lung (P1 and P2) and brain (P14). Red arrows indicate lesion sites. (**B**) The frequency of the *Nocardia* species found in patients by metagenomic next-generation sequencing. The number of circles denotes the case count for each detected *Nocardia* species.

Radiological findings varied across patients. The most frequent abnormalities on chest CT included exudative shadow, nodular shadow, consolidation shadow, cavitations, and pleural effusions. Detailed imaging findings are shown in Figure 1A. Twelve patients (63.2%) had bilateral lung involvement.

### 3.2. Microbiological Findings

In this study, 27 samples performed with both culture and mNGS were obtained from 19 patients. The specimens formed wrinkled colonies on the culture medium surface, accompanied by the “agar-biting phenomenon” (Figure S1A). After 72 h of incubation, Gram-stained sputum specimens containing thin, branched mycelia were observed (Figure S1B). This colony morphology represents a characteristic feature of *Nocardia* culture. Using mNGS, 11 different *Nocardia* of species level were found. *Nocardia farcinica* was the most frequently occurring species (*n* = 11), followed by *Nocardia cyriacigeorgica* (*n* = 3), *Nocardia abscessus* (*n* = 2), and *Nocardia brasiliensis* (*n* = 2) (Figure 1B).

Seven (36.84%) *Nocardia* positive cases were identified by both mNGS and culture. Twelve (63.16%) cases were confirmed as positive by mNGS alone. Using clinical diagnosis as reference, the sensitivity and specificity of mNGS for the diagnosis of *Nocardia* infections in patients with suspected infections can be up to 100% (Supplementary Table S2). The sensitivity of mNGS was significantly higher than that of culture (100% vs. 36.84%, *p* < 0.001).

**Figure 2:**
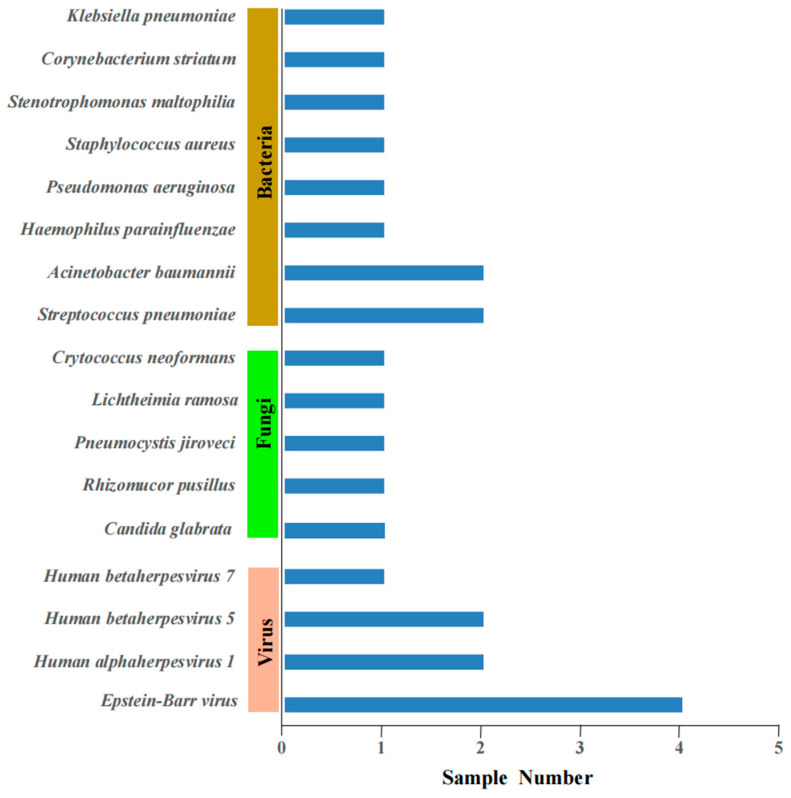
Other microorganisms detected by metagenomic next-generation sequencing (mNGS) are shown in three categories: bacteria, fungi, and viruses.

In addition to *Nocardia* species, mNGS also identified other microorganisms from the same clinical samples (Figure 2). Viruses had been found in nine patients, including *Epstein–Barr virus* and *Human herpesvirus*. Other bacteria have also been found in four patients, including *Acinetobacter baumannii*, *Klebsiella pneumoniae*, and *Streptococcus pneumoniae* (Figure 2). Combined with clinical manifestations and conventional culture methods, these viruses and other bacteria were considered as colonization or unlikely pathogens. Fungi were found in six patients, including *Rhizomucor pusillus*, *Lichtheimia ramosa*, *Pneumocystis jiroveci*, *Candida glabrata*, and *Cryptococcus neoformans* detected by mNGS and *Aspergillus* spp. detected by the GM test. In recent years, there have been emerging reports suggesting the potential for colonization by *Pneumocystis jirovecii* [12]. *Candida* is a common component of the normal human microbiota throughout the body; however, the clinical significance of detecting *Candida* species in the respiratory tract is becoming increasingly uncertain [13]. In conjunction with clinical manifestations and serological tests, *Rhizomucor pusillus*, *Cryptococcus neoformans*, *Lichtheimia ramosa*, and *Aspergillus* spp. were identified as co-infections with *Nocardia* in four (21.05%) patients (Supplementary Table S1).

### 3.3. Treatment and Outcome

**Figure 3:**
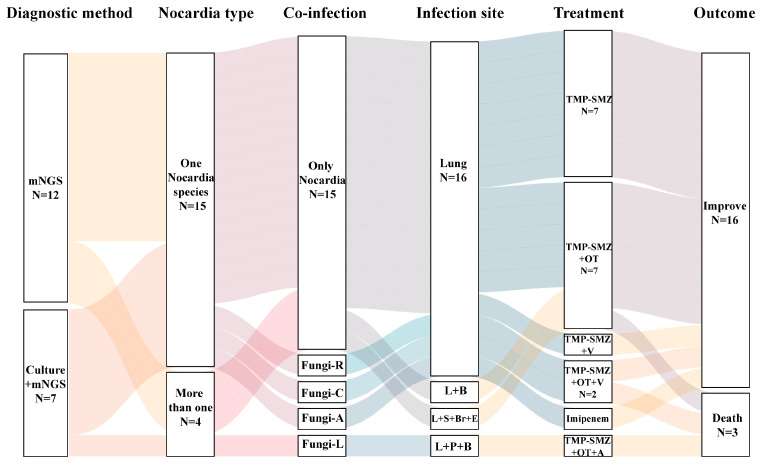
Treatment strategies for all patients by Sankey diagram. B: blood, Br: brain, E: eye, L: lung, P: pleural, S: skin, Fungi-R: *Rhizomucor pusillus*, Fungi-C: *Cryptococcus neoformans*, Fungi-A: *Aspergillus* spp., Fungi-L: *Lichtheimia ramosa*, OT: other drugs for *Nocardia*, A: amphotericin B, V: voriconazole.

All 19 patients were diagnosed with *Nocardia* infection based on mNGS and/or culture results and received antibiotic therapy. The antimicrobial regimens used are summarized in Figure 3 and Supplementary Table S1. Apart from *Nocardia*, fungi were also found by mNGS and considered as co-infection in four patients. Trimethoprim-Sulfamethoxazole (TMP-SMZ) was the most commonly used treatment of *Nocardia* infection. In some cases, TMP-SMZ was combined with other antibiotics such as carbapenems, amikacin, or linezolid. For patients with fungal co-infections, voriconazole was administered in three cases and amphotericin B in one.

Among the 19 patients, 16 (84.2%) showed clinical improvement or were cured. Three patients (15.8%) died after receiving treatment for 10 to 38 days. Two of the three patients who died were found to be co-infected with fungal infections-*Aspergillus* spp. and *Lichtheimia ramosa*. Voriconazole and amphotericin B were used as antifungal agents, respectively. The mortality rate for co-infected fungal infections was 50% (2/4), while the mortality rate for only *Nocardia* infections was 6.7% (1/15, *p* = 0.097). The main causes of death in patients were poor control of the underlying diseases and severe suppression of immune function.

## 4. Discussion

This study found that pulmonary nocardiosis predominantly affects immunocompromised individuals with underlying conditions. However, the clinical and imaging characteristics are nonspecific. The application of mNGS can improve the diagnostic rate for *Nocardia*. This combined approach positively influences prognosis and intervention outcomes. TMP-SMZ, either alone or in combination with other agents, remains the primary treatment, and the overall prognosis is generally favorable.

The isolation of *Nocardia* from respiratory tract specimens or other body sites often indicates infection. This is because *Nocardia* is a saprophytic bacterium widely distributed in soil, water, air, and decaying vegetation. It is not part of the normal human flora and rarely causes laboratory contamination. Notably, recent studies have reported that a positive *Nocardia* culture does not inherently signify infection, as colonization remains a potential interpretation [14,15,16]. Therefore, diagnosis of *Nocardia* infection requires comprehensive evaluation, including clinical symptoms, imaging findings, immune status, and treatment response. In this study, all 19 cases underwent professional evaluation, with final clinical diagnoses of pulmonary nocardiosis confirmed. *Nocardia* can form mycelium in the air that is then inhaled through the respiratory tract, making the lung the most susceptible organ to infection. It can also infiltrate skin wounds or spread by hematogenous dissemination, and can even induce meningitis or brain abscess [17,18]. In this study, we found that three cases showed dissemination to multiple sites, including the lungs, bloodstream, pleural cavity, skin, brain, and eyes.

Nocardiosis typically occurs in individuals with impaired cellular immunity, including those receiving long-term corticosteroids, patients with malignancies, transplant recipients, and those on immunosuppressive therapy. Immunodeficient patients have an acute or subacute onset, with high fever, and are prone to disseminated infections in the bloodstream and other elsewhere, resulting in a high mortality rate. Chronic lung diseases such as COPD and bronchiectasis have also been found to be risk factors for pulmonary nocardiosis in recent years [19]. The risk of *Nocardia* infection also exists in individuals with healthy immune systems and no underlying disease [19]. In this study, 89.47% (17/19) of cases had underlying diseases, including four cases of pulmonary underlying diseases such as bronchiectasis and COPD, and 14 cases had systemic diseases, long-term oral glucocorticoids, and/or other immunosuppressive drugs. In addition, 68.43% (13/19) of cases had fever, including hyperthermia in six cases. Imaging findings of *Nocardia* infection lacked specificity. Masses and cavities are common in immunodeficient patients, while solid, nodular, and ground glass shadows are predominant in immunocompetent patients [20]. In our cohort, 63.16% (12/19) had bilateral lung involvement, and exudates, solid lesions, nodules, cavities, and pleural effusions were frequently observed, which is consistent with the aforementioned literature.

The most common tests for *Nocardia* infection are smear microscopy and culture. The gold standard for diagnosis of *Nocardia* infection is to culture specimens from their respiratory secretions, abscess needle aspirates, biopsy tissue, and other suitable samples. Direct microscopy can quickly yield results, but its drawback is a low positive rate. Gram staining and weak acid-fast staining are positive, which can easily lead to misdiagnosis as *Mycobacterium* [21,22]. The culture cycle is long, and colony formation generally takes approximately 3 days. The detection or diagnosis rate of *Nocardia* has increased recently due to the clinical focus on the disease and improvement in identification methods, such as matrix-assisted laser desorption ionization-time of flight mass spectrometry (MALDI-TOF MS) and 16S rRNA gene sequencing, particularly the use of mNGS techniques [23,24,25,26]. MALDI-TOF MS identification inherently relies on pure microbial cultures. The accuracy of identification is profoundly shaped by the completeness of proteomic databases and the timeliness of database maintenance [27,28]. 16S rRNA gene sequencing allows rapid identification of bacteria without relying on cultures. The use of MALDI-TOF MS for initial identification and 16S rRNA gene sequencing for ambiguous ones may be a viable cost-effective approach. Nevertheless, there is a subset of strains that can only be identified to the genus level. Compared to traditional culture methods, mNGS provides a faster turnaround time and higher sensitivity in the detection of *Nocardia* [11,29]. In this study, the culture method alone identified only seven positive cases, representing a sensitivity of 36.84%. However, the mNGS successfully identified *Nocardia* species in all patients, representing a sensitivity of 100%. The sensitivity of mNGS for detecting *Nocardia* was significantly higher than that of culture (*p* < 0.001). Furthermore, mNGS could identify *Nocardia* to species level. There are 173 species of *Nocardia* according to the most recent taxonomy of the genus (https://lpsn.dsmz.de/, accessed on 9 March 2025), 54 of which are associated with human infections, such as *N. farcinica*, *N. asteroides*, *N. brasiliensis*, and *N. abscessus* [30,31,32]. *N. farcinica* and *N. cyriacigeorgica* are the most frequently isolated species, which are widely distributed in China [33]. In this study, *N. farcinica* and *N. cyriacigeorgica* showed the highest detection rates, similar to the abovementioned literature data. It is suggested that mNGS has a high sensitivity for the detection of *Nocardia* species and can be a powerful complement to culture. When clinical suspicion of *Nocardia* infection is raised, multiple methods, such as culture and mNGS, should be sent as soon as possible.

Patients with weakened immune systems are more susceptible to mixed infections [34]. Owing to its unbiased nature, mNGS is capable of detecting diverse microorganisms within a single sample. This study found that 4 of 19 patients had co-infections with other pathogens, namely *Rhizomucor pusillus*, *Cryptococcus neoformans*, *Lichtheimia ramosa*, and *Aspergillus* spp. The study revealed that mixed infections, especially those combined with fungal infections, have poor prognoses. The mortality rate for co-infected fungal infections was 50%, while the mortality rate for only *Nocardia* infections was 6.7% (*p* = 0.097). Three patients passed away as a result of inadequate control of the underlying diseases, significant immune function suppression, and ineffective treatment. Therefore, early detection and treatment are crucial to reducing patient mortality. We used mNGS combined with conventional laboratory-based diagnostic testing to gain more accurate and comprehensive information related to co-infecting microbes in patients. mNGS offers the important benefit of detecting co-infected pathogens.

The type of bacteria, and the patient’s immune status, the location, extent, and severity of the lesion, and drug sensitivity tests should all be taken into consideration when developing an empirical anti-infective treatment plan for nocardiosis. TMP-SMZ, which exhibits high blood concentration, excellent tissue permeability, and the ability to cross the blood–brain barrier into the central nervous system, is the drug of choice for treating *Nocardia* infections. Other commonly used drugs include imipenem or meropenem, amikacin, linezolid, cephalosporins, quinolones, and minocycline. Combination therapy is necessary for serious illness, disseminated infections, or cerebral infections. The prognosis of pulmonary nocardiosis is related to the patient’s immune status and the promptness of treatment, with a mortality rate of approximately 14–40% [19,21,35]. In our study, TMP-SMZ was used as the preferred treatment drug, including 8 cases of monotherapy and 10 cases of combination therapy, with a cure and improvement rate of 84.2%.

There are some limitations. Firstly, as a single-center retrospective study, it is potentially prone to bias. Future multicenter, prospective, or case-control studies are needed to further evaluate the clinical value and cost-effectiveness of mNGS in diagnosing pulmonary nocardiosis [36,37]. Secondly, due to the limited number of culture-positive cases, there is a dearth of drug susceptibility data. Considering the significant variations in drug susceptibility among different *Nocardia* species and the latest classification system that divides clinically relevant *Nocardia* species into 13 antimicrobial susceptibility patterns [38], it is recommended that drug susceptibility tests be performed using the microbroth dilution method (MIC) on cultured strains to inform treatment plans.

## 5. Conclusions

In conclusion, misdiagnosis is common due to the nonspecificity of clinical and imaging presentations of pulmonary nocardiosis. The timely combination of mNGS represents a promising approach to enhance the diagnosis of pulmonary nocardiosis and inform targeted antimicrobial therapy. TMP-SMZ is the first line of treatment.

## Figures and Tables

**Table 1:** Clinical characteristics of 19 patients with pulmonary nocardiosis.

Characteristic	Mean ± SD, or N (%)
Age (years)	58 ± 15
Gender	
Male	11 (57.9)
Female	8 (42.1)
Underlying diseases	
Acute leukemia	4 (21.1)
Multiple myeloma	1 (5.3)
Bronchiectasis	2 (10.5)
Chronic obstructive pulmonary disease (COPD)	2 (10.5)
Antineutrophil cytoplasmic antibody-associated vasculitis	2 (10.5)
Diabetes mellitus type 2	2 (10.5)
Systemic lupus erythematosus	1 (5.3)
Dermatomyositis	1 (5.3)
Membranous nephropathy	1 (5.3)
Hemolytic anemia	1 (5.3)
None	2 (10.5)
Immunosuppressive therapy	14 (73.7)
White blood cell (WBC), 10^9^/L	9.07 ± 7.59
Lymphocyte count (LY), 10^9^/L	1.22 ± 1.78
Serum albumin (ALB), g/L	32.48 ± 9.15
Clinical symptoms	18 (94.7)
Cough	11 (57.9)
Sputum	8 (42.1)
Fever	13 (68.4)
No symptom	1 (5.3)
Isolated pulmonary nocardiosis	16 (84.2)
Disseminated course, extrapulmonary foci	3 (15.8)
Brain	1 (5.3)
Skin	1 (5.3)
Blood	2 (10.5)
Pleura	1 (5.3)
Eye	1 (5.3)
Imaging features	
Exudative shadow	11 (57.9)
Nodular shadow	8 (42.1)
Consolidation shadow	6 (31.6)
Cavity formation	4 (21.1)
Pleural effusion	7 (36.8)

Note: SD: standard deviation, N: number.

## Data Availability

The data presented in this study are available on request from the corresponding author due to patient privacy.

## Supplementary Materials

The following supporting information can be downloaded at: https://www.mdpi.com/article/10.3390/pathogens14070656/s1, Table S1: Information of 19 patients with pulmonary nocardiosis; Table S2: Performance results of mNGS and culture in the diagnosis of pulmonary nocardiosis; Figure S1: The morphology of *Nocardia* culture.
