# Supplementary material for: Clinical Features of Pulmonary Nocardiosis and Diagnostic Value of Metagenomic Next-Generation Sequencing: A Retrospective Study
Source: Pathogens. 2025 Jul 2;14(7):656. doi: 10.3390/pathogens14070656 (PMC12301029; doi:10.3390/pathogens14070656)
Supplement: Supplementary file 1 [file pathogens-14-00656-s001.zip › Supplementary Figure S1.pdf]

Supplementary Figure

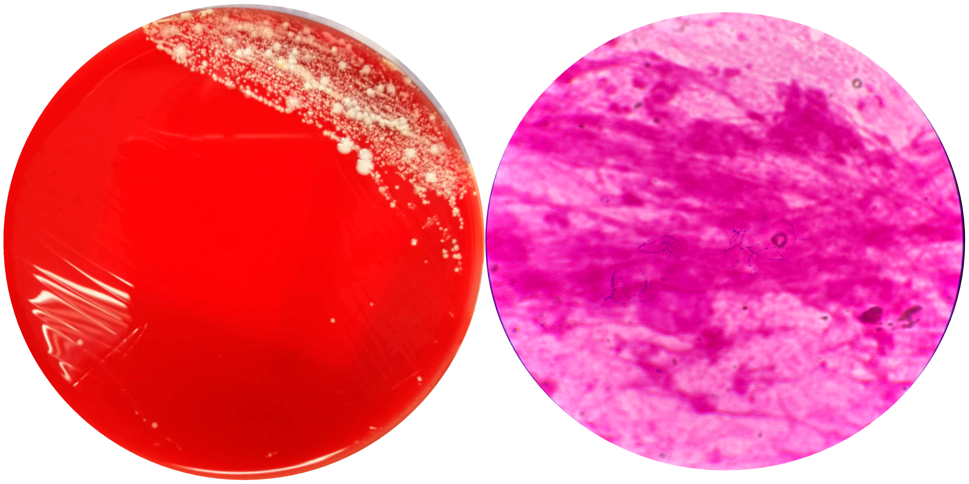

(**A**) (**B**)

Figure S1. The morphology of *Nocardia* culture. (**A**) Colony morphology diagram. The diameter of the petri dish is 9 cm. The specimens formed wrinkled colonies on the culture medium surface, accompanied by the "agar—biting phenomenon". (**B**) Gram staining microscopy image. Magnification is 1000×. After 72 hours of incubation, Gram-stained sputum specimens containing thin, branched mycelia were observed.
